# How to manage antipsychotic-induced akathisia

**DOI:** 10.1192/j.eurpsy.2021.1287

**Published:** 2021-08-13

**Authors:** N. Moura, D. Esteves-Sousa, J. Facucho-Oliveira, C. Laginhas, A. Quintão

**Affiliations:** 1 Psychiatry Department, Ocidental Lisbon Hospital Center, Lisboa, Portugal; 2 Psychiatry, Hospital de Cascais, Cascais, Portugal

**Keywords:** Akathisia, Antipsychotics, extrapyramidal, Anxiety

## Abstract

**Introduction:**

Akathisia is a relatively common adverse effect of antipsychotics although some second-generation antipsychotics are known to have a lower liability for the condition. The core feature of akathisia is mental unease characterized by a sense of agitation, usually accompanied by motor restlessness, which can cause patients to pace up and down and be unable to stay seated for more than a short time. An association between this discomfiting subjective experience and suicidal ideation has been postulated but remains uncertain.

**Objectives:**

Our aim is to perform a non-systematic review of the literature regarding the current understanding of antipsychotic-induced akathisia and its management.

**Methods:**

A semi-structured review was conducted on Pubmed concerning the relationship between akathisia and antipsychotics.

**Results:**

All antipsychotics drugs can cause akathisia. The management of antipsychotic-induced akathisia should include a dose reduction of the antipsychotic treatment or a switch to quetiapine or olanzapine. If ineffective, a trial with propranolol may be useful as well as the addition of a 5-HT_2A_ antagonist like mirtazapine or mianserine. At last the inclusion of a benzodiazepine may be helpful albeit the risk of dependence and anticholinergics mainly when other extrapyramidal symptoms are present.

**Conclusions:**

High‐dose antipsychotic medication, antipsychotic polypharmacy and rapid increase in antipsychotic dosage should be avoided to prevent akathisia. There is limited evidence for any pharmacological treatment for akathisia such as switching to an antipsychotic medication with a lower liability for the condition, or adding a beta‐adrenergic blocker, a 5‐HT_2A_ antagonist or an anticholinergic agent although some patients may benefit from such interventions.

